# Therapeutic Target Identification and Drug Discovery Driven by Chemical Proteomics

**DOI:** 10.3390/biology13080555

**Published:** 2024-07-23

**Authors:** Mingjie Zou, Haiyuan Zhou, Letian Gu, Jingzi Zhang, Lei Fang

**Affiliations:** State Key Laboratory of Pharmaceutical Biotechnology, Chemistry and Biomedicine Innovation Center, Medical School of Nanjing University, Nanjing 210093, China; 502023350059@smail.nju.edu.cn (M.Z.); 502023350056@smail.nju.edu.cn (H.Z.); 211230020@smail.nju.edu.cn (L.G.); zhangjingzi@nju.edu.cn (J.Z.)

**Keywords:** chemical proteomics, small molecule, target identification, drug discovery, off-target

## Abstract

**Simple Summary:**

Human beings are always associated with small molecules throughout their lives. Chemical proteomics, which selectively identifies small-molecule targets, is an emerging approach for unraveling the mechanism of action of small molecules for therapeutic target identification and drug discovery. This review attempts to introduce representative techniques of chemical proteomics and summarizes examples of identifying small-molecule targets.

**Abstract:**

Throughout the human lifespan, from conception to the end of life, small molecules have an intrinsic relationship with numerous physiological processes. The investigation into small-molecule targets holds significant implications for pharmacological discovery. The determination of the action sites of small molecules provide clarity into the pharmacodynamics and toxicological mechanisms of small-molecule drugs, assisting in the elucidation of drug off-target effects and resistance mechanisms. Consequently, innovative methods to study small-molecule targets have proliferated in recent years, with chemical proteomics standing out as a vanguard development in chemical biology in the post-genomic age. Chemical proteomics can non-selectively identify unknown targets of compounds within complex biological matrices, with both probe and non-probe modalities enabling effective target identification. This review attempts to summarize methods and illustrative examples of small-molecule target identification via chemical proteomics. It delves deeply into the interactions between small molecules and human biology to provide pivotal directions and strategies for the discovery and comprehension of novel pharmaceuticals, as well as to improve the evaluation of drug safety.

## 1. Introduction

**Figure 1:**
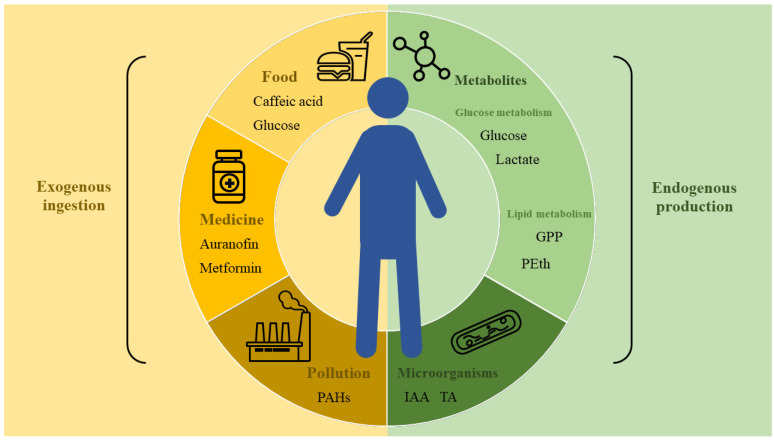
Multiple small molecules regulating the human homeostatic system. The human organism incorporates exogenous small molecules from dietary intake, pharmaceutical interventions, and environmental exposure. Simultaneously, endogenous small molecules are synthesized through glucose and lipid metabolic pathways, as well as secretions from the internal microbiome. This review elucidates several exemplary small molecules, demonstrating their collective role in modulating physiological functions and pathological processes within the body.

Undoubtedly, from conception to demise, the human life cycle is inextricably linked with small molecules. The small molecules, based on their origins, can be broadly categorized into exogenous and endogenous types, both of which synergistically regulate bodily functions and disease manifestation (Figure 1). For instance, caffeic acid, commonly found in tea beverages, has been shown to ameliorate inflammatory responses [1]. Conversely, polycyclic aromatic hydrocarbons, emitted from industrial activities, possess the potential to induce hepatotoxicity [2]. Endogenous small molecules encompass not only those produced through human metabolism, such as carbohydrates and nucleosides, but also regulatory factors generated by the microbiota. The precise equilibrium among the myriad of endogenous molecules is crucial for maintaining homeostasis [3]. It is worth noting that the distinction between exogenous and endogenous molecules is not always clear-cut; some compounds, like lactate [4] and glucose [5], can be both ingested from external sources and synthesized within the body.

When the homeostatic balance of the body is disrupted by disease or external factors, endogenous metabolites initially respond, regulating their synthesis and degradation to compensate for the organism’s function [6,7]. However, when homeostatic imbalance exceeds the body’s metabolic capacity, exogenous small-molecule drugs are required for intervention. Some synthetic small molecules interact with specific protein targets by mimicking or modulating endogenous metabolite activity, thereby regulating bodily functions. Additionally, naturally active small-molecule drugs can also intervene in or adjust specific molecular mechanisms and biological pathways within the body, albeit with more complex structures and less elucidated mechanisms of action. Therefore, promoting drug discovery and exploring the molecular mechanisms of disease occurrence can provide more precise guidance for disease prevention, diagnosis, and treatment.

The process of drug discovery is inherently complex, with a history spanning millennia. In initial phases of drug discovery, researchers are confronted with the critical challenge of identifying and validating potential therapeutic targets [8]. The therapeutic targets not only modulate disease progression but also possess potential druggability, offering unique opportunities for pharmaceutical development. Therapeutic targets can be classified into druggable and undruggable categories. Druggable targets typically feature deep groove structures that can accommodate low molecular weight, “drug-like” small molecules, thereby facilitating therapeutic effects [9]. An example is HER2, which is regarded as an efficacious therapeutic target in breast cancer treatment [10]. In contrast, undruggable targets present difficulties for drug binding due to structural limitations, such as the absence of accessible deep hydrophobic pockets or active sites [11]. The representative proteins in this group include transcription factors like STAT3, TP53, and MYC [12]. However, advancements in targeting methodologies have enabled the transformation of previously undruggable targets into druggable ones. A notable illustration of the shift is the mutant oncogene KRAS, long considered undruggable. Recent discoveries have revealed its druggability, with inhibitors targeting KRAS (G12C), such as AMG510 and MRTX849, demonstrating promising therapeutic outcomes in clinical trials [13].

Target safety and efficacy remain as paramount concerns in drug discovery [14]. A recent analysis indicates that over half of lead compounds are terminated due to preclinical toxicity, with safety considerations in phase I and II clinical trials being a significant factor [15]. One contributing factor is the occurrence of off-target effects in systemically administered drugs. Off-target effects refer to the significant impact of drug molecules on drug safety and efficacy by interacting with multiple proteins due to the similar binding sites or structural features, thereby significantly impacting drug safety and therapeutic efficacy [16]. A notorious example is thalidomide, initially used to alleviate pregnancy-related nausea but later found to cause fetal malformations and multiple birth defects [17]. To address such issues, target-oriented drug discovery approaches are essential. The methods for target identification focus more intently on the molecular mechanisms of diseases, aiming to enhance target selectivity and affinity while reducing non-target interference. By identifying and validating therapeutic targets that modulate disease progression, the approaches can substantially mitigate the impact of off-target effects.

In this regard, the target identification method based on affinity chromatography has appeared as early as the 1960s and has been extensively utilized [18,19,20]. Additionally, researchers have developed various identification and analysis strategies, including transcriptome-wide compound feature spectrum analysis [21], chemical genomics approaches [22], and yeast two-hybrid methods [23]. Nonetheless, the techniques are plagued by issues such as false positives [24] and limited applicability [25]. As a high-throughput and reliable technique for identifying molecular targets in the post-genomic era, chemical proteomics has found widespread application in target identification, drug discovery, and various other fields [26]. Chemical proteomics enable the high-throughput identification of covalent or non-covalent protein targets of small molecules both intracellularly and in vivo, facilitating the identification of potential off-target sites and comprehensive characterization of therapeutic targets. At the same time, chemical proteomics is not limited to the identification of recombinant proteins but can be used to probe entire proteomes or defined sub-proteomes. Furthermore, compared with traditional methods, the application scope of chemical proteomics is broader and can be applied to any cell type or tissue from numerous species, ranging from humans to microorganisms [27]. At present, through chemical proteomics technology, we can more comprehensively understand the structure and function of proteins and explore the potential interactions between proteins and small molecules.

In this review, we briefly discuss the significant implications of chemical proteomics in the identification of small-molecule targets for targeted therapeutic efficacy and the promotion of preclinical drug discovery. Additionally, we integrate chemical proteomics for safety assessment to address the potential off-target effects of drugs, aiming to ensure sustainable drug discovery.

## 2. The Methods in Chemical Proteomics

### 2.1. Probe-Based Chemical Proteomics

The prevalent strategy in chemical proteomics is predicated on the utilization of chemical probes to enrich molecular targets from cell or tissue lysates, commonly referred to as probe-based chemical proteomics. This approach encompasses three fundamental steps: (1) the design and synthesis of the probes; (2) the capture and separation of targets using affinity chromatography; and (3) the identification of targets by mass spectrometry (MS) [28].

Chemical probes constitute a potent class of instrumental molecules that are employed for fluorescence imaging and molecular enrichment within the domain of chemical biology. Among these, affinity probes are a subset of chemical probes that possess handles for affinity purification and serve a pivotal role in target discovery mediated by chemical proteomics [29].

#### 2.1.1. Classification of Chemical Probes

##### Immobilized Probes

Active small molecules are covalently conjugated to biologically inert matrices, such as agarose and magnetic beads. These solid matrices facilitate the enrichment of targets for subsequent identification. Nevertheless, the convenience of immobilized matrices is counterbalanced by the challenge of high spatial resistance, which can lead to the loss of targets with weak binding affinity [28]. These probes may also inadvertently enrich proteins with affinity to the immobilized matrices or linkers, and the establishment of negative controls can help to eliminate false positive results [30,31].

##### Activity-Based Probes

With the continuous development of the field, scientists have developed activity-based probes (ABPs) to replace defective immobilized probes [32]. These ABPs incorporate reporter groups such as biotin for enrichment [33] and fluorescent groups for detection [34]. Streptavidin, known for its high affinity for biotin, is effectively utilized for the enrichment of target proteins [35]. However, the drawbacks associated with the typically large and hydrophobic reporter groups attached to conventional ABPs may modify the activity of the compound molecules and impede intracellular in situ binding. The azide-alkyne cycloaddition (AAC) is a foundational reaction in click chemistry [36]. Upon the binding of the compound molecule to the protein, the reactive group forms covalent bonds with amino acid residues at the protein’s active site. Subsequently, the click chemistry reaction is employed to conjugate an enrichment tag or tracing tag for enrichment or detection. Typically, azide is used to label the enrichment tag, while alkyne groups are used to label the compound molecule [37,38]. These probes can directly bind to the target in situ within living cells due to the retention of the compound’s intrinsic activity, thereby providing a more accurate depiction of small molecule–protein interactions [39]. Nevertheless, the direct attachment of the reporter group to the small molecule may lead to a reduction in activity and modification of cellular processes [40]. Furthermore, the enrichment process may be influenced by the cell’s endogenous biotin. The initial click chemistry reaction, which relies on Cu ion catalysis, can be toxic to cells and may compromise in situ binding. The refined strain-promoted alkyne-azide cycloaddition (SPAAC) employs a cyclooctyne ring structure to expedite the reaction [41,42]. Due to the ring structure of the “click” handle, the steric hindrance effects between probes and proteins become larger. It creates a new problem. Furthermore, certain natural compound molecules are bound non-covalently to target proteins, and click chemistry may disrupt non-covalent secondary bonds, potentially resulting in off-target effects.

##### Photoaffinity Probes

Photoaffinity probes represent an advanced iteration of ABPs, which is predicated on the concept of photoaffinity labeling (PAL) [43]. To circumvent the potential disruption of non-covalent interactions by click chemistry, photoreactive groups, such as benzophenone, aryl azides, and diazirines, are integrated into the click chemistry probes [44]. Upon binding to the target protein and upon subsequent activation with wavelength-specific light (typically ultraviolet light at 365 nm), these probes release highly reactive chemicals that covalently cross-link proximal amino acid residues, effectively converting non-covalent interactions into covalent ones. This is subsequently followed by the click chemistry process as previously described, which safeguards against the exclusion of non-covalently bound target proteins during enrichment. The primary limitation of photoaffinity probes is the occurrence of non-specific binding in the photoreactive groups. A comparative study examining the non-specific binding of three distinct photoreactive moieties revealed that the non-specific binding is structurally selective [45]. This finding can be utilized to refine the specificity of photoaffinity probes.

#### 2.1.2. Workflow of Probe-Based Chemical Proteomics

**Figure 2:**
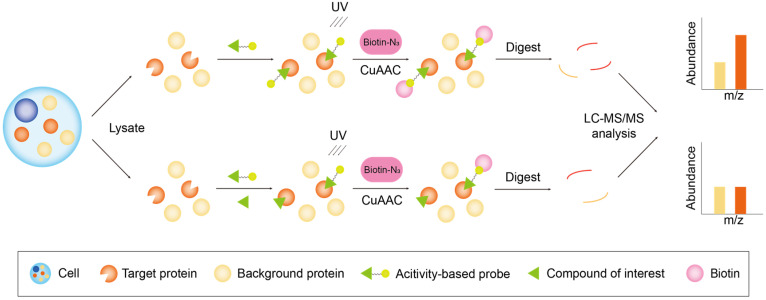
The figure depicts the workflow of ABPP. ABPs are engineered to interact with a target protease at its active site. The top route involves the co-incubation of the cellular proteome with ABPs, which engage with the target protein at its active site and subsequently form a covalent bond between the probe’s small molecules and the target protein’s macromolecules through PAL. This bond ensures the stable association of the probe with the target protein. The ABPs can then be conjugated with biotin through a copper (I)-catalyzed azide-alkyne cycloaddition (CuAAC)-catalyzed click reaction, enabling their subsequent pull-down using the affinity of streptavidin for biotin. The proteins are then processed into peptides for subsequent MS analysis. The bottom route describes the steps of competition groups. Native small molecules are used to compete with the ABPs for the active site of the target protein. The proteins binding to the competition molecules are excluded from affinity purification and, as a result, a weaker signal is observed. By comparing the relative signal strengths in the two routes, the target proteins can be identified with high confidence. The orange columns denote proteins potentially capable of binding to small molecules, while yellow columns signify background proteins.

**Figure 3:**
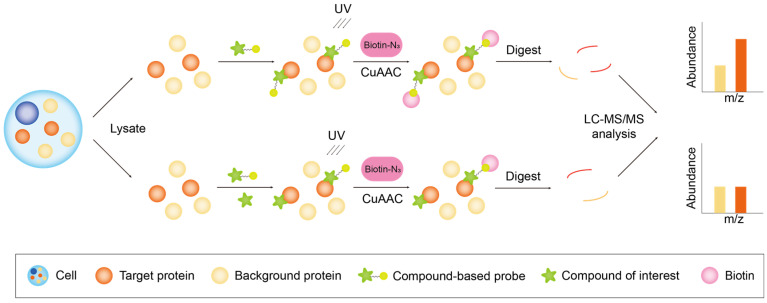
The figure depicts the workflow of CCCP. The probe is crafted to complement the structure of the compound of interest, introduced into the proteome, and conjugated to its target protein. It forms a covalent bond with the target protein through an electrophilic trap or a photocrosslinking group. Samples undergo a CuAAC-catalyzed click chemistry reaction to conjugate an affinity tag (biotin), which is subsequently used for the enrichment of the tagged peptides, facilitating a downstream analysis through the affinity of streptavidin for biotin. The orange columns denote proteins potentially capable of binding to small molecules, while yellow columns signify background proteins.

Target identification based on probe-driven methods can be broadly classified into two distinct workflows: (1) activity-based protein profiling (ABPP), which concentrates on the enzymatic activities of specific protein families (Figure 2); and (2) compound-centric chemical proteomics (CCCP), which delves into the molecular mechanisms of action for individual biologically active small molecules (Figure 3) [25].

##### Activity-Based Protein Profiling

ABPP was introduced in 1999 [46], and subsequently, its potential for target identification was explored and refined. With advancements in affinity probe synthesis technology, ABPP has evolved into a prominent method for the identification of the small-molecule targets of compounds. The core of ABPP lies in the affinity probe, which is typically composed of three distinct components: the reactive moiety, the linker, and the reporter moiety. ABPP purifies proteins by establishing a covalent bond between the affinity probe and the protein, with the reactive moiety facilitating the attachment of enrichment labels [14] (Figure 2). Following incubation of the probe with cell lysate, the probe–target complex is conjugated to biotin through click chemistry, followed by the enrichment of the complex using streptavidin beads. The proteins are then directly digested into peptides using trypsin or eluted from the streptavidin beads and subsequently digested into peptides. Protein identification is achieved through MS; as the protein target cannot bind to the probe following incubation with the compound, the principle of competitive binding can be utilized to directly identify the compound’s target [47].

##### Compound-Centric Chemical Proteomics

Unlike ABPP methods, which primarily rely on the covalent or non-covalent binding of a small molecule substrate to an enzyme, CCCP methods provide a more comprehensive coverage of the binding of a small molecule to its target proteins, based on strong affinity interactions. Strong affinity is predicated on the structural complementarity between the small molecule and the target protein, and the binding event can influence the structure and activity of the protein, thereby elucidating the mechanism of action. CCCP integrates affinity purification with MS techniques, leveraging the robust affinity between small molecules and target proteins (Figure 3). The traditional CCCP method involves the synthesis of the probe by covalently immobilizing the small molecule onto a specific solid substrate, typically agarose beads or resin. Subsequently, cell lysates are incubated with the probes within the solid matrix. Subsequently, two steps of elution are conducted: the first step removes proteins that do not bind to the probes; and the second step isolates the target proteins. The target proteins are then analyzed and identified using proteomics methods [48,49]. More recently, the CCCP workflow has been refined and adapted to align with that of ABPP, employing affinity-based probes for target protein binding and click chemistry for biotin addition, facilitating streptavidin-mediated enrichment [50,51,52].

Both methods facilitate high-throughput, unbiased target identification, each with their distinct advantages and limitations. The high-throughput nature of these techniques is underpinned by the application of protein MS techniques. Within the cellular proteome, there are often numerous proteins capable of binding to the small molecules of compounds. In the application of chemical proteomics, the affinity-purified proteins are initially enzymatically digested into peptides by trypsin, with each peptide then being analyzed by MS. This process enables the concurrent analysis of multiple target proteins that bind to small molecules in a single batch.

The enzymatic activity-based feature of ABPP enables the identification of proteins in their active conformations. Conversely, CCCP capitalizes on the robust binding of compound molecules to target proteins for direct protein enrichment, which is considered a more balanced methodology. Nevertheless, ABPP’s specific reliance on the active site of binding proteins restricts its application to proteins with enzymatic activity.

### 2.2. Probe-Free Chemical Proteomics

Each of the aforementioned chemical proteomics methods necessitates the design and synthesis of probes, a process that typically involves a considerable investment of time and effort [29]. Furthermore, modifying the compound molecule or conjugating a substrate may affect its conformation and solubility, potentially introducing bias into the results. Additionally, certain natural compounds may lack suitable modification sites, hindering their synthesis as probes [28]. Consequently, in response to these limitations, researchers have developed a suite of probe-free chemical proteomics methods that depend on the biophysical variations in the conformations of proteins for the identification of molecular targets.

#### 2.2.1. Thermal Proteome Profiling

**Figure 4:**
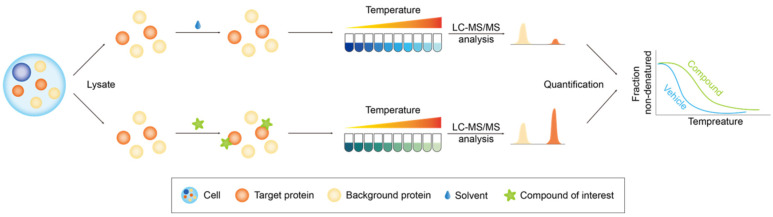
The figure depicts the workflow of TPP. Following incubation of the proteome with the compound of interest or a solvent, the proteome was subjected to a temperature gradient treatment to induce protein precipitation, and the subsequent supernatants were isolated. Subsequently, the supernatants were processed for protein quantification through MS, and protein melting curves were generated. Ultimately, the proteins with a propensity to bind small molecules were delineated through a comparative analysis of the quantitative data obtained from the two distinct cohorts. The orange columns represent proteins prone to bind small molecules, whereas yellow columns signify background proteins.

The stability of proteins is significantly improved when they are bound to small molecules [53]. The cellular thermal shift assay (CETSA) employs the alteration in the characteristic melting temperature (T_m_) and melting curve to pinpoint protein targets [54,55]. Cell lysates are incubated with compound small molecules and subsequently heated, leading to the precipitation of unbound proteins with reduced thermal stability due to heat-induced denaturation. Following centrifugation to remove the precipitated proteins, the remaining soluble proteins are quantified using protein detection techniques such as Western blotting, and melting curves are generated. Thermal proteome profiling (TPP), also referred to as MS-CETSA, integrates MS-based quantitative proteomics for protein identification and quantification, enabling proteome-wide protein stability mapping within physiologically relevant environments (Figure 4). In contrast to the conventional CETSA, TPP facilitates the high-throughput identification of soluble target proteins by turning to MS for protein quantification. It is essential to recognize that even if the binding of small molecules to large molecular weight proteins induces a conformational change, the extent of this alteration may not be sufficient to induce a substantial change in thermal stability, and precipitation may still occur upon heating, potentially leading to false-negative outcomes [56].

#### 2.2.2. Drug Affinity Responsive Target Stability

The drug affinity responsive target stability (DARTS) assay, developed by Lomenick [57], is predicated on the principle that proteins display differential susceptibility to hydrolytic enzymes in the presence and absence of small-molecule compounds. The general methodology involves the co-incubation of the small-molecule compound with cell lysate, followed by the introduction of protein hydrolase. Following cessation of the enzyme reaction, the proteins within the gel are fractionated and isolated using SDS-PAGE and subsequently analyzed by quantitative MS [58].

#### 2.2.3. Stability of Proteins from Rates of Oxidation

Protein stability, modulated by ligand binding, encompasses resistance to oxidative and chemical denaturation. The stability of proteins from rates of oxidation (SPROX) assesses protein stability by quantifying the rate of oxidation of methionine residues under varying concentrations of chemical denaturants to identify targets [59]. Because SPROX specifically targets peptides with exposed methionine residues, it may overlook a significant amount of potential target information. The recently developed chemical denaturation and protein precipitation (CPP) [60] and solvent-induced protein precipitation (SIP) [48] methods have been developed to address the limitations of SPROX in terms of protein coverage.

#### 2.2.4. Target-Responsive Accessibility Profiling

**Figure 5:**
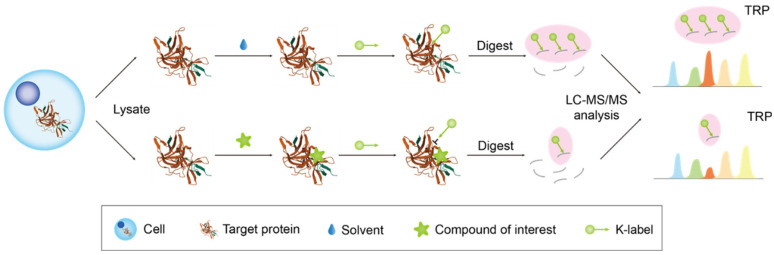
The figure depicts the workflow of TRAP. The TRAP labeling method, which involves reductive dimethylation modification of lysine residues in the proteome using CD_2_O in combination with BPC, leads to a mass shift in the lysine residues. Subsequently, the enriched proteins were identified through MS, and the responsive proteins were categorized as target candidates by comparing the differences in protein ion intensities between the control and experimental groups for screening purposes. The orange columns denote target-responsive peptides (TRPs), whereas the remaining columns represent background peptide.

In 2023, Tian proposed a method known as target-responsive accessibility profiling (TRAP) (Figure 5) [61]. The underlying principle is that upon binding of the compound of interest to the target protein, the active lysine residues in the corresponding binding region become inaccessible for covalent labeling due to spatial site blocking or metastable effects. Consequently, TRAP labeling was executed using isotope-coded formaldehyde (CD_2_O) in conjunction with a borane–pyridine complex (BPC), which results in a mass shift of +32.06 Da for the lysine residues, circumventing interference from naturally occurring lysine dimethylation. Subsequently, MS was conducted, and the results were analyzed by comparing the alterations in accessibility of proteasome labelable proteins (i.e., differences in the labeling of lysine residue dimethylation) between samples treated with and without ligands. The concept of the TRAP ratio was introduced, denoting the quantitative ratio of lysine-containing peptides. Peptides exhibiting significant alterations in TRAP ratios were designated as target-responsive peptides (TRPs), and the associated proteins were classified as potential candidate targets.

Tian subsequently validated the method using two pairs of small molecules known to bind to known target proteins. They first employed purified ribonuclease A (RNase) in conjunction with its ligands cytidine diphosphate (CDP) and cytidine triphosphate (CTP). Following TRAP treatment, the labeling intensity at lysine 41 near the RNase binding site was notably attenuated upon treatment with CDP and CTP, whereas the labeling intensities at the more distant lysines 91 and 104 remained unchanged. Another pair of a target protein and its ligand, pyruvate kinase M2 (PKM2) and its allosteric activator fructose 1,6-bisphosphate (FBP), also validated the method’s feasibility through the TRAP ratio. Furthermore, the researchers utilized the TRAP method within a cellular system, illustrating that potential false-positive results could be eliminated through measured response experiments. These steps corroborated the capability of TRAP to identify targets within complex cellular settings.

In summary, probe-free chemical proteomics circumvents the potential disruption of small-molecule activities during the probe synthesis process, leading to substantial cost-efficiency. Nevertheless, the current absence of effective enrichment techniques poses a challenge to the identification of low-abundance proteins, potentially resulting in a high frequency of false-negative results. In this context, probe-based chemical proteomics offers high throughput, sensitivity, and broad protein coverage. We provided a comparative overview of probe-based and probe-free chemical proteomics in Table 1. Consequently, we conclude that TRAP is currently the most effective target screening technique. Therefore, in the future, the advancement of more sensitive instruments and the introduction of innovative research methods may help alleviate some of the limitations of current techniques.

### 2.3. Quantitative Proteomics

Traditional methods for protein purification, which rely on gel electrophoresis technology, are characterized by limitations in separation accuracy and sensitivity. Quantitative proteomics represents an innovative chemical proteomics approach that mitigates the non-specific binding issues endemic to earlier affinity-based methods, thereby substantially enhancing the precision of target identification [24]. Quantitative proteomics is commonly categorized into three primary approaches: label-free quantitative proteomics, chemical labeling methods, and stable isotope labeling with amino acids in cell culture (SILAC).

SILAC is recognized as the most prevalent quantitative proteomics approach for target identification. It operates on the principle that the cellular proteome is labeled with isotopes by repeatedly culturing cells in medium supplemented with isotope-labeled amino acids. Target proteins are then enriched using active probes, and the molecular weights obtained by MS are compared with those of a control group to discern alterations in the relative abundance of proteins. Thereby identifying the binding proteins of the small-molecule compounds [62,63].

The quantitative proteomics methods that rely on chemical labeling encompass the isotope-coded affinity tag (ICAT), tandem mass tag (TMT), and isobaric tag for relative and absolute quantification (iTRAQ). Among these, iTRAQ is the most prevalent chemical labeling method [64]. In contrast to SILAC, which is limited to the analysis of three samples per LC-MS/MS run, iTRAQ technology enables the simultaneous comparison of up to eight samples, thereby significantly enhancing throughput [65,66]. Furthermore, SILAC is unsuitable for studies involving primary tissue samples due to the requirement to label the cellular proteome, a process that inherently includes cell passaging. iTRAQ circumvents this constraint by directly labeling proteins chemically.

Label-free quantitative proteomics employs MS to analyze enzymatically cleaved peptides from proteins utilizing LC-MS. This approach is favored for its cost-efficiency. In this method, the MS data generated during the large-scale identification of proteins is analyzed, and the signal intensities of the corresponding peptides in different samples are compared to quantify the proteins. Label-free quantitative proteomics encompasses two primary approaches: spectral counting and intensity-based quantification. The former involves the enumeration and comparison of the number of fragment ion spectra (MS2) for peptides of a specific protein. The second approach, intensity-based quantification, entails the measurement of the MS signal intensity [67]. Nevertheless, this method exhibits limitations in terms of accuracy and throughput when compared with quantitative labeling proteomics methods. Specifically, in complex proteomes with heterogeneous backgrounds, maintaining accuracy can be a significant challenge [68].

### 2.4. MS Acquisition Schemes

MS serves as a pivotal platform within chemical proteomics, and the method employed for data acquisition significantly influences the quality of subsequent analysis. Currently, the prevailing data acquisition strategies in MS are data-dependent acquisition (DDA) and data-independent acquisition (DIA). DDA represents an established data acquisition approach. The underlying principle involves performing primary MS analysis and subsequently selecting parent ions for secondary MS analysis based on predefined criteria, thereby enabling the acquisition of additional fragmentation information. This approach employs a narrower selection window to target ions, which can minimize interference ions to a certain degree. Nevertheless, this approach may inadvertently select ions with high peak intensities as target ions for secondary MS analysis, potentially leading to inaccurate sampling and reduced analytical reproducibility. Consequently, DDA is less appropriate for the analysis of complex samples due to these limitations [14]. As an extension and refinement of the DDA approach, the DIA approach primarily acquires all MS at the first level by utilizing alternating high and low collision energies, eliminating the need for parent ion selection. Subsequently, a series of scanning windows are employed for the full-range scanning of first level MS, facilitating a rapid, cyclic identification and detection of all ions within each window. In summary, it is feasible to capture all fragmentation information for all ions in the sample, enhancing data utilization, minimizing missing data, bolstering analytical reproducibility, and making DIA particularly well-suited for the analysis and detection of large and complex samples [69].

## 3. Target Identification of Small Molecules

### 3.1. Target Proteins of Small Metabolite Molecules and Their Analogs

Small metabolite molecules, as crucial factors in maintaining the diversity and complexity of human life activities, have been increasingly linked to various diseases. Recent studies have revealed connections between gut microbiome metabolites, serum metabolites, urinary metabolites, and numerous diseases, including kidney disorders [70], heart diseases, and neurodegenerative ailments [71]. The metabolites interact with biological processes by targeting proteins, altering local or global structures of proteins, and they regulate bodily homeostasis [72]. The interaction between small metabolite molecules and proteins typically occurs through non-covalent binding or covalent post-translational modifications. Non-covalent binding involves metabolites interacting with proteins via hydrogen bonds, ionic bonds, and van der Waals forces, influencing protein structure and function. For instance, adenosine triphosphate (ATP) binds non-covalently to kinases, modulating protein activity for cellular signal transduction [73]. Conversely, metabolites with reactive functional groups can form covalent modifications on target proteins when encountering nucleophilic residues like cysteine and lysine. Examples include lipidation [74], sulfoxidation [75], and glycosylation [76], all of which have been shown to regulate protein function. Therefore, understanding small metabolite molecules-induced changes in protein structure and function within dynamic cellular environments is a core challenge in modern biology research and drug discovery.

In recent years, chemical proteomics platforms have made significant strides. The advancements have not only enhanced the efficiency and accuracy of small metabolite molecules target identification but have also provided researchers with deeper insights into metabolite–biomolecule interactions [77]. This section mainly focuses on the latest developments and applications of chemical proteomics in identifying targets for the small molecules of lipid and carbohydrate metabolism (Table 2 and Table 3), aiming to facilitate the characterization of small-molecule drug candidates with clinical relevance.

#### 3.1.1. Small Molecules of Lipid Metabolism

As essential cellular components with diverse functions, lipids form cell membrane structures, modify proteins post-translationally, and regulate signaling pathways [89]. Lipidation, a post-translational modification, allows for direct interaction with cell membranes. It is one of the most widespread modifications in nature [90]. Common types include fatty acylation, prenylation, and glycosylphosphatidylinositol anchoring [91]. Lipidation enhances protein lipophilicity, enabling interactions with membranes and membrane-bound proteins. Consequently, lipidated proteins participate in various cellular processes, including signal transduction, cell adhesion, and protein transport. Moreover, lipids can non-covalently bind target proteins through specific lipid-binding domains or recognition modules. The interaction is based on affinity between lipids and proteins. For instance, lipids can directly interact with membrane-bound proteins like lipoprotein-binding proteins, altering protein structure and function to modulate membrane properties and intercellular interactions [92].

Currently, over a thousand structurally diverse lipids have been identified in biological systems. The lipids target proteins in various ways, regulating crucial cellular signaling pathways. However, the complex network poses challenges for elucidating molecular mechanisms and drug discovery. Traditional methods for detecting protein lipidation and prenylation have involved metabolic labeling with radioactive fatty acids and isoprenoid precursors, followed by immunoprecipitation [90]. But the approach was time-consuming, produced weak signals, and posed radiation risks, making it unsuitable for routine use [91]. Non-radioactive methods like acyl-biotin exchange also have limitations [93]. Recent innovations in chemical proteomics have allowed for a global analysis of lipid–protein interactions. It is achieved through metabolic labeling using functionalized lipid analogs modified with azide or alkyne groups [94]. For instance, interferon-induced transmembrane proteins (IFITMs), important host antiviral defense factors, regulate viruses like influenza A virus (IAV), Ebola virus (EBOV), and severe acute respiratory syndrome coronavirus (SARS-CoV) [95,96,97]. Das et al. synthesized a bifunctional cholesterol analog for proteomic and targeted protein photocrosslinking studies [78]. Combined with molecular dynamics simulations, they found that cholesterol directly interacts with S-palmitoylated IFITMs in cells, altering the conformation in the membrane bilayer and affecting host susceptibility to different viruses. Isoprenoid pyrophosphates participate in protein prenylation and cellular regulation. To discover the proteins involved in isoprene pyrophosphate interactions, Cai et al. developed a chemical proteomics strategy using a desulfo-biotin-geranyl pyrophosphate (GPP) acyl phosphate probe and SILAC [79]. They discovered numerous candidate GPP-binding proteins and, for the first time, found that histone deacetylase 1 (HDAC1) interacts with GPP. Phospholipase D (PLD) transphosphatidylation activity leads to alcohol consumption and the formation of phosphatidylethanol (PEth), a biomarker for chronic alcoholism [98]. Yu et al. used PLD’s bifunctional primary alcohol to generate a clickable photoaffinity phosphatidyl alcohol probe [80]. It allowed for the visualization of phosphatidyl alcohol generation sites and comprehensive understanding of phosphatidyl alcohol interactions with the cellular proteome. They revealed an interaction with the single-channel transmembrane protein basigin/CD147, providing insights into the potential pathophysiological role of PEth.

#### 3.1.2. Small Molecules of Carbohydrate Metabolism

Carbohydrates serve as the primary energy source for the human body. They undergo oxidative breakdown to generate and maintain energy, playing a crucial role in metabolic processes. Carbohydrates metabolism occurs through aerobic and anaerobic pathways, with cells selecting the appropriate route based on specific needs and environmental conditions [99]. Recent studies suggest that small molecules of carbohydrate metabolism may function beyond mere metabolic fuel. They potentially act as important signal messengers and versatile regulatory factors. However, the precise mechanisms of the interaction with binding targets and functional regulation remain largely elusive [100]. Chemical proteomics has emerged as a novel method for identifying endogenous metabolite targets, effectively addressing the challenge. A recent study by Miao et al. utilized azide-glucose click probes combined with chemical proteomics to investigate glucose [101]. They discovered that glucose could act as a signaling molecule, directly binding to and dimerizing DDX21, an RNA helicase associated with tissue differentiation, thereby regulating epidermal differentiation. Furthermore, chemical proteomics has facilitated new explorations into the non-metabolic functions of other small molecules of carbohydrate metabolism. The approach contributes to a deeper understanding of how small metabolite molecules transcend the traditional energy and structural roles, participating in complex cellular functions and physiological processes.

In aerobic conditions, pyruvate from glycolysis enters mitochondria for further oxidation to acetyl-CoA. The process culminates in the tricarboxylic acid cycle, where complete oxidation yields water and carbon dioxide. Concurrently, oxidative phosphorylation generates substantial ATP. Acetyl-CoA, a pivotal metabolic intermediate, serves as a convergence point for carbohydrate, lipid, and protein metabolism within cells. Levy et al. developed CATNIP (CoA/AcetylTraNsferase Interaction Profiling), a chemical proteomics platform for analyzing acyl-CoA/protein interactions [87]. The innovative approach employs quantitative LC-MS/MS to identify acetyl-CoA binding proteins without bias and examines various protein–CoA metabolite interactions, and it utilizes a system-level analysis to evaluate the characteristics of protein networks potentially interacting with acyl-CoA. Levy et al. demonstrated the practicality of CATNIP by exploring acetyl-CoA/protein interactions in the proteome of HeLa cells. Chemical proteomics data from competitive binding assays using 0, 3, 30, and 300 μM acetyl-CoA were transformed, revealing eight protein clusters. Various clusters exhibit distinct dose-dependent competitive characteristics. But in clusters 1–3, acetyl-coenzyme A was observed to antagonize protein capture in a dose-dependent manner. Clusters 1 and 2 encompassed direct acetyl-CoA binding proteins (e.g., CREBBP), indirect binding proteins (e.g., NAA25), and allosteric proteins (e.g., PANK1). Cluster 3 contained a significant number of mitochondrial CoA-binding enzymes. Notably, protein capture capability was completely lost at concentrations exceeding 30 μM. In essence, CATNIP integrates chemical proteomics and systems biology analyses to provide high-confidence annotations of direct acetyl-CoA-binding proteins. The approach enhances our understanding of the crucial roles played by acyl-CoA metabolic pathways in biological processes and disease states. ATP, the primary energy carrier in organisms, binds to numerous functional cellular proteins. Some act as key regulators in cell signaling, such as kinases. Abnormal kinase signaling contributes to various human diseases, including cancer [102]. In this regard, Wang et al. developed a clickable ATP photoaffinity probe for the global analysis of ATP-binding proteins [81]. To minimize interference with protein binding, they synthesized a novel ATP probe by attaching a photoreactive diazirine group and an alkyne handle to the terminal phosphate ester of ATP, and the novel probe was synthesized via SN2 reaction. Wang et al. employed the innovative ATP probe to label HEK293T cell lysates, incorporating a biotin tag and utilizing a “click” reaction with rhodamine azide. Subsequent LC-MS/MS analysis resulted in the high enrichment of 3031 proteins, including 582 annotated ATP-binding proteins and 249 kinases. Notably, lower concentrations of the ATP probe exhibited enhanced specificity, leading to the improved enrichment of ATP-binding proteins. The ATP probe design demonstrated superior kinase enrichment capabilities compared with previous methods. Furthermore, the probe shows potential for analyzing other ATP-competitive drugs and identifying novel ATP-binding proteins.

Under anaerobic conditions, the citric acid cycle is impeded. Pyruvate from glycolysis accumulates due to excess nicotinamide adenine dinucleotide (NADH), and lactate dehydrogenase then catalyzes pyruvate conversion to lactate, regenerating NAD^+^. Th process is known as anaerobic fermentation. Research indicates that rapidly dividing cells exhibit an increased glucose metabolism to support proliferation, resulting in a high lactate concentration [103]. To investigate whether lactate accumulation directly impacts the proliferative state, Liu et al. utilized TPP to identify direct lactate target proteins in human embryonic kidney cells [88]. They discovered that accumulated lactate inhibits the active site of the SUMO protease SENP1 and leads to the structural remodeling of the ana-phase-promoting complex/cyclosome (APC/C) that binds to ubiquitin-conjugating enzyme E2 C (UBE2C), thus regulating the degradation of cell cycle proteins and the exit from mitosis in HeLa S3 and HCT116 cells. The approach marks the first elucidation of lactate’s direct regulatory mechanism on protein function to control the cell cycle and proliferation. NAD^+^ serves as an electron carrier and acts as a coenzyme for numerous dehydrogenases in the body, playing a crucial role in key pathways such as glycolysis and gluconeogenesis. NAD^+^ also serves as a substrate for signal transduction enzymes mediating post-translational modifications [104]. However, enzymes utilizing NAD^+^ as a substrate can cleave its nicotinamide glycosidic bond, resulting in NAD^+^ depletion, such as poly-ADP-ribose polymerases (PARPs 1−17). Therefore, Sileikyte et al. developed clickable PAL probes to analyze potential NAD (NAD^+^/NADH) interactomes [82]. To ensure the enzyme stability of the nicotinamide glycosidic bond, the researchers selected benzamide adenine dinucleotide (BAD), an NAD^+^ analog capable of inhibiting PARP-1- and NAD^+^/NADH-binding enzymes. Exploiting the solvent-exposed or partially exposed N-6 and C-2 positions on BAD’s adenine ring, they synthesized BAD analogs 2-ad-BAD and 6-ad-BAD by attaching linkers containing a diazirine and terminal alkyne at the positions. Using the clickable PAL NAD probes, the team analyzed the NAD interactome in HEK 293T cell lysates, identifying both known and previously unknown NAD/NADH binding proteins. The approach offers potential for elucidating NAD interactomes in various disease contexts. However, spatial constraints or ineffective photocrosslinking with the 2- and 6-ad-BAD probes may have resulted in an incomplete identification of NAD binding proteins, thus requiring further investigation.

Hyperactivated glycolysis serves as a metabolic hallmark for majority of cancer cells [105]. Cancer cells exhibit increased aerobic glycolysis dependence through metabolic reprogramming, even in oxygen-rich conditions. Glucose breakdown releases lactate to meet elevated synthetic metabolic demands for cancer cell proliferation. The phenomenon is commonly referred to as the “Warburg effect” [106]. In this regard, Hao et al. pioneered TRAP chemical proteomics method to measure ligand binding-induced changes in target recognition accessibility via the global labeling of reactive protein lysine. The accessibility response target profiling was completed for 10 glycolytic metabolites in the HCT116 colorectal cancer cell line (FBP, F6P, G6P, R5P, G3P, 2PG, 3PG, PEP, Pyr, Lac) and identified 913 candidate targets [61]. The research revealed multiple potential regulatory modes of glycolytic metabolites, deepening the understanding of their role as signaling molecules coordinating crucial pathways in cancer cells.

#### 3.1.3. Other Small Metabolite Molecules

Choline, a constituent of biological membranes and precursor of acetylcholine in cholinergic neurons, plays a role as a metabolic intermediate in the Kennedy pathway and potentially characterizes cancer occurrence [107]. A recent chemical proteomics analysis by Dixit et al. elucidated choline metabolite interactions and specific target activities [83]. Utilizing structurally diverse choline analogs as metabolic probes, they discovered phosphocholine’s ability to inhibit the anticancer target p32 and its binding to various interacting partners, thereby enhancing anticancer activity. Bile acids (BAs) primarily exist in the enterohepatic circulation system, defending against pathogens, inhibiting bacterial overgrowth, and regulating gut microbiota composition. However, some pathogenic bacteria exhibit BA tolerance [108,109]. Liu et al. addressed the issue using a photoaffinity BA probe based on primary bile acid cholic acid (CA) combined with quantitative chemical proteomics [84]. They identified potential BA-interacting proteins in *Escherichia coli* (*E. coli*), notably the histidine kinase EnvZ from the bacterial two-component system. BA binds to EnvZ, forming specific structures and transducing signals downstream, promoting the bacterial expulsion of excess BA. Therefore, EnvZ can act as a novel BA tolerance sensor in bacteria, surviving in high BA concentrations.

#### 3.1.4. Interactions between Microbe and Host

Microorganisms, Earth’s most abundant biological resource, colonize various human body sites, including the skin, gastrointestinal tract, and respiratory system. They form complex, discrete ecosystems, adapting to each ecological niche’s environmental conditions [110]. Thus, microbial imbalance can impair metabolic and immune functions, jeopardizing human health. Recent studies reveal that both microbial properties and their metabolites influence host physiology [111]. For example, *E. coli* converts tryptophan into indole-containing metabolites, acting as signaling molecules to regulate host physiology, alleviate intestinal inflammation, and prevent diseases such as inflammatory bowel disease [112]. *Prevotella*, predominant in semen, secretes uromodulin with immunosuppressive and anti-inflammatory properties [113]; *Lactobacillus* in vaginal and cervicovaginal regions produces antimicrobial substances, resisting bacterial and viral infections [114]. Therefore, deciphering molecular processes at host–microbe interfaces facilitate novel therapeutic strategies and reveals new targets for disease development. Chemical proteomics, a powerful tool for metabolite target identification, has successfully elucidated microbial metabolite mechanisms, advancing host–microbe communication research [115,116,117].

The small metabolites molecules secreted by human microbiota can alter protein structure and function through non-covalent binding and covalent post-translational modifications, regulating metabolic pathways. Microbes produce millimolar concentrations of short-chain fatty acids, such as butyrate, which modulate host metabolism and anti-inflammatory capabilities through protein acylation. However, the specific immune mechanisms remain elusive. Zhang et al. designed alkyne butyrate analogs and employed chemical proteomics to detect target proteins in *Salmonella* typhimurium regulated by *Salmonella* pathogenicity island-1 (SPI-1) [85]. The results revealed butyrate’s ability to directly acylate microbial virulence factors like HilA, inhibiting pathogenicity in vivo; regarding non-covalent interactions, gut microbiota metabolizes dietary aromatic amino acids into bioactive molecules such as indole-3-acetic acid (IAA) and tryptamine (TA). They participate in specific receptor and signaling pathways by directly binding to target proteins [118,119,120]. To define protein targets and determine precise mechanisms, Zhao et al. developed a chemical proteomics method based on IAA and TA photoaffinity probes [86]. Method efficacy was demonstrated in identifying microbial indole metabolite-interacting proteins, particularly orphan G protein-coupled receptors (GPCRs), facilitating the development of small-molecule agonists for orphan receptors.

### 3.2. Annotation on the Target of Small-Molecule Drugs

Demand for novel therapeutic strategies in medicine and pharmaceuticals continues to grow, driving an urgent need for innovative drug targets [27]. However, elusive mechanisms of action and limited specificity hinder further clinical application. There are more than 20,000 possible protein targets for drug discovery in humans, but only about 600 human proteins targets have had corresponding FDA-approved molecule drugs until 2016 [121] and approximately averaged 10 novel targets approved by the United States, European Union, and Japan annually between 2019 and 2023 [122,123,124,125,126].

Currently, discovered drugs can be broadly categorized into large and small-molecule drugs based on molecular weight. Large molecule drugs, such as blocking antibodies and vaccines, typically exceed 1000 Daltons and require injection or infusion [127]. Despite therapeutic benefits, the drugs face challenges like poor tissue penetration and transmembrane transport, affecting the efficacy and risking immune-related adverse events [128,129]. Small-molecule drugs, usually below 1000 Daltons, easily penetrate cell membranes as exogenous specific substances. They directly interact with target proteins, ensuring pharmacological activity. Additionally, small molecules offer cost advantages in manufacturing, storage, transportation, and administration compared with large molecules [130]. Thus, fully annotating specific targets of small-molecule drugs helps describe the close connection between pharmacological effects and cellular components, avoiding off-target effects. In this review, we mainly focus on the latest developments and applications of chemical proteomics in annotating the targets of both synthetic and natural small-molecule drugs (Table 4 and Table 5). By identifying protein targets and analyzing uncharacterized interactions, the annotation of the drug’s intrinsic functional targets is accomplished.

#### 3.2.1. Synthetic Small-Molecule Drugs

Throughout history, scientists have designed and synthesized numerous small-molecule drugs with specific functions by delving into the pathological pathways and key proteins within organisms. The drugs function by inhibiting or activating the activity of target proteins, thus exerting their pharmacological effects. Some of the early synthetic molecule drugs, such as aspirin (acetylsalicylic acid) and phenytoin sodium, are still in use today, while some others have disappeared from the market but have played a significant role in advancing treatment approaches for specific indications [131]. The development of synthetic small-molecule drugs has provided a powerful tool for precision medicine, enabling personalized treatment based on the specific genetic backgrounds and disease states of patients [132]. Their small-molecule nature ensures good cell permeability and bioavailability, facilitates large-scale production, and ensures quality control, providing a reliable guarantee for clinical application. Chemical proteomics offers a more precise understanding of the interactions between synthetic drugs and their targets, thereby optimizing the drug design and screening process.

**Table 4:** Probe-based target identification of small-molecule drugs.

Small Molecule	Target Method	Target Protein	Probe Structure
Metformin	Photoactive metformin probe [133]	Presenilin enhancer 2 (PEN2)	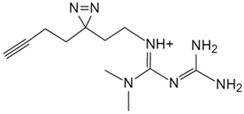
Baicalin	Photoaffinity baicalin probe [134]	Carnitine palmitoyltransferase 1 (CPT1)	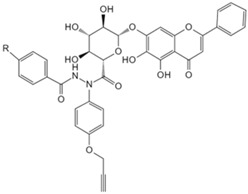
Berberine	Biotinylated berberine probe [135]	Pyruvate kinase isozyme type M2 (PKM2)	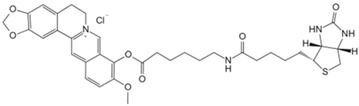

**Table 5:** Probe-free based target identification of small-molecule drugs.

Small Molecule	Target Method	Target Protein
Auranofin	Thermal proteomic profiling (TPP) [136]	Thioredoxin reductase 1 (TXNRD1)
Methotrexate	Cellular thermal shift assay (CETSA) [137]	Phosphoglycerate kinase 1 (PGK1)
Triptolide	TPP based on XGBoost (X-TPP) [138]	Heterogeneous nuclear ribonucleoprotein A2/B1 (HnRNP A2/B1)

Auranofin, an oral medication for rheumatoid arthritis, reduces rheumatoid factor and antibody formation, and research reveals auranofin has potent anti-tumor activity [139,140,141]. Saei et al. conducted thermal proteome profiling on human colorectal cancer cells, combining expression proteomics and redox proteomics [136]. The results confirmed thioredoxin reductase 1 (TXNRD1) as auranofin’s primary target, confirming that its mechanism closely relates to redox enzyme pathway disruption. Methotrexate, an anti-folate antineoplastic drug, primarily inhibits dihydrofolate reductase, impeding tumor cell synthesis, growth, and proliferation [142]. Current research focuses on metabolism and downstream pathways. Zhang et al. employed quantitative proteomics to screen for methotrexate-resistant target proteins [137]. Using CETSA, they demonstrated methotrexate’s interaction with phosphoglycerate kinase 1 (PGK1) in vitro and in vivo, identifying PGK1 as a novel target and providing valuable reference for PGK1’s inhibitor design. Metformin, widely used for type II diabetes treatment, exhibits anti-aging and anti-cancer properties. Clinical use spans nearly 60 years. Studies indicate a close association with AMP-activated protein kinase (AMPK) [143,144], yet direct molecular targets remain elusive. Ma et al. overcame chemical synthesis challenges, creating photosensitive metformin probes [133]. Through multi-step screening, they identified PEN2 (a γ-secretase subunit) as a key binding protein. Ma et al. discovered that PEN2 intersects with lysosomal glucose-sensitive pathways via ATP6AP1. The PEN2–ATP6AP1 axis mediates metformin’s AMPK activation, clarifying therapeutic benefits and offering potential targets for metformin alternative screening.

#### 3.2.2. Natural Small-Molecule Drugs

Over the past three decades, the search for efficient and low-toxicity lead compounds from natural products has been a focal point in new drug research [28]. Compared with chemically synthesized drugs, natural products and their derivatives have significant advantages in structural diversity and unique activity. Statistics reveal that more than 50% of drugs approved by the United States Food and Drug Administration (FDA) in the past are natural drugs, such as resveratrol, curcumin, and artemisinin, greatly impacting human health and disease progression [145]. However, the complexity of chemical components in most natural products, requiring certain concentration ranges and appropriate ratios for synergistic action, makes elucidating their mechanisms challenging. Chemical proteomics enables a holistic study of all proteins expressed by cell or tissue genome using natural small-molecule drugs, analyzing their dynamic changes and activity patterns to promote drug discovery.

Baicalin, a flavonoid compound extracted from dried roots of *Scutellaria baicalensis*, exhibits therapeutic effects against hepatitis, nephritis, and allergic diseases, and has anti-steatosis properties [146,147]. A recent quantitative chemical proteomics analysis by Dai et al. revealed baicalin’s ability to allosterically activate carnitine palmitoyl transferase 1 (CPT1), accelerating fatty acid degradation [134]. The mechanism significantly improves hepatic steatosis-related symptoms and reduces diet-induced obesity, offering potential for metabolic disorder treatments. Berberine, a quaternary ammonium alkaloid isolated from *Coptis chinensis*, demonstrates antibacterial properties and metabolic improvements in colorectal cancer cells and also promotes apoptosis and inhibits cell proliferation [148,149]. To study the direct target proteins of berberine, Yan et al. synthesized biotinylated berberine and integrated chemical proteomics to identify the direct target protein PKM2 in colorectal cancer cells [135]. They found that berberine regulates PKM2 ubiquitination and degradation and suppresses metabolic reprogramming in the cells, thus proving its potential as a cancer treatment candidate. Tripterine is a kind of diterpene trioxide extracted from the root bark of *Tripterygium wilfordii* with various biological activities, exhibiting antioxidative, anti-rheumatic, and anticancer effects [150,151,152]. Utilizing the TPP method trained with the XGBoost machine learning algorithm, namely X-TPP, Chen predicted all potential binding targets of triptolide based on protein thermal stability curves [138]. They reported for the first time the potential interaction between triptolide and the mRNA-binding protein heterogeneous nuclear ribonucleoprotein A2/B1 (HnRNP A2/B1) and indicate that triptolide exerts its anticancer effects by inhibiting the HnRNP A2/B1-PI3K/AKT pathway. Furthermore, the novel chemical proteomics approach offers a novel and effective target screening strategy for other natural products with complex chemical components.

## 4. Evaluation of the Off-Target Effects of Drugs

Currently, widespread clinical use of certain drugs occurs despite the incomplete annotation of their targets. Consequently, numerous medications interact with multiple targets, inevitably leading to side effects and drug resistance during treatment [153]. Industry-standard analysis strategies primarily rely on large-scale screening combinations using various purified enzymes to address potential off-target issues [26]. However, the approach may not comprehensively cover all potential off-target effects due to its dependence on specific enzyme assays. Additionally, in vitro experiments fail to fully reflect drug behavior in vivo. Chemical proteomics emerges as a novel strategy for identifying potential off-target effects. Efficient and comprehensive omics analyses and the exploration of drug–protein interactions in near-physiological conditions make chemical proteomics a powerful tool for modern drug design and biomedical research.

Orlistat, or tetrahydrolipstatin (THL), an anti-obesity drug approved by the U.S. FDA with potential anti-tumor activity, has a high global demand. To further explore the cellular off-target scenarios of THL as much as possible, Yang et al. introduced highly conservative modifications into the parent THL structure, combined with bio-orthogonal click chemistry [154]. They designed a probe that retains THL-like activity for target identification. Fatty acid synthase, a protein crucial for cancer cell growth, was identified as an anticipated target of THL. Yang et al. also revealed eight other new potential off-target proteins for THL. Identifying the targets could explain the various pharmacological actions or side effects of THL, aiding in the early development stages of THL-like drugs in cancer treatment. Fatty acid amide hydrolase (FAAH), a membrane enzyme that terminates fatty acid amide-class signaling lipids, has seen the development of inhibitors for treating central nervous system diseases. However, a particular FAAH inhibitor, BIA 10-2474, was found to potentially cause off-target effects, leading to human neurotoxicity events [155,156,157]. In response, Huang et al. used an alkyne-modified BIA 10-2474 metabolite probe and click chemistry-ABPP and discovered that BIA 10-2474’s demethylated metabolite could covalently modify conserved catalytic cysteine residues of aldehyde dehydrogenases [158]. The residues often participate in pathways protecting the brain from oxidative stress damage. Thus, the results indicate that BIA 10-2474 and its metabolites have off-target sites inhibiting human nervous system functions, causing metabolic dysregulation in the nervous system.

However, the off-target effects of drugs can sometimes benefit patients, leading to the discovery of new pharmacological properties and a reevaluation of the drug’s function. For example, Jackson [159] reported that subjects experienced penile erection while taking sildenafil, which was originally used to treat angina pectoris. The side effect of the drug can be used to treat sexual dysfunction, and sildenafil has therefore changed its indication. Thus, leveraging beneficial off-target effects can introduce innovative and promising approaches to precision medicine. By identifying specific patient groups’ unique responses to certain drugs, physicians can tailor more accurate and safer treatment plans based on the individual conditions and needs of patients.

## 5. Conclusions

The identification of drug targets remains a significant technical bottleneck in drug discovery and chemical biology research [160]. Developing accurate and high-throughput target identification methods is crucial for accelerating drug discovery, reducing associated costs and unveiling opportunities for novel therapeutic agents. While this review has highlighted numerous advancements and successes in chemical proteomics-based target identification, the impact of the analyses on drug discovery is still constrained by mass spectrometry technology due to cost and requisite expertise. Nevertheless, it is anticipated that as the sensitivity and resolution of mass spectrometry analysis and quantification continue to improve, coupled with advancements in data acquisition and analysis algorithms, the target identification capabilities of chemical proteomics will become increasingly precise. Furthermore, the integration of chemical proteomics with other molecular network levels, such as metabolomics and transcriptomics, will expand the scope of dynamic protein functional mapping and provide a comprehensive systemic view of drug action [49].

Looking ahead, chemical proteomics approaches that integrate diverse technological strengths are expected to broaden their target identification coverage. Chemical proteomics will likely capture subtle changes in global proteomics studies, facilitating a deeper understanding of molecule–target interactions and supporting clinical research. The progression will contribute to the thorough elucidation of pharmacological and toxicological mechanisms, providing essential research support for addressing challenges in new drug discovery and the identification of human disease biomarkers.

## Figures and Tables

**Table 1:** Comparison of target identification methods.

Method	Advantages	Disadvantages
ABPP	1. Binds to the active site 2. Reveals enzyme activity	1. Probe synthesizing is cumbersome 2. Support for chemical synthesis required
CCCP	1. Targets proteins based on strong affinity 2. Extensive screening available	
TPP	1. No synthetic probes required 2. Simple process	1. Low resolution; 2. Unable to locate binding site
TRAP	1. No synthetic probes required 2. Simple process 3. High resolution 4. Locate the binding site in peptide	

**Table 2:** Probe-based target identification of small metabolite molecules.

Small Molecule	Method	Target	Probe Structure
Cholesterol	Cholesterol photoaffinity probe [78]	S-palmitoylated Interferon-induced transmembrane proteins	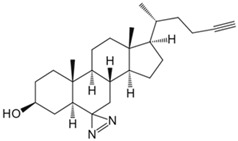
Geranyl pyrophosphate	Desthiobiotin-Geranyl pyrophosphate acyl phosphate probe [79]	Histone deacetylase 1	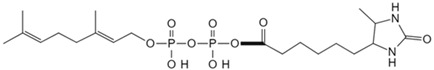
Phosphatidylethanol	Clickable photoaffinity phosphatidyl alcohol probe [80]	Basigin/CD147	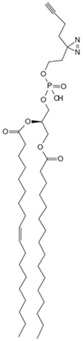
Adenosine triphosphate	Clickable Adenosine triphosphate photoaffinity probe [81]	Adenosine triphosphate-binding proteins	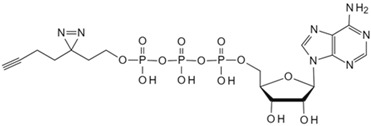
Nicotinamide adenine dinucleotide	Photoaffinity 2-ad-BAD probe [82]	Nicotinamide adenine dinucleotide-binding proteins	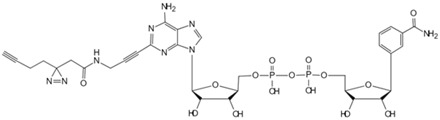
Nicotinamide adenine dinucleotide	Photoaffinity 6-ad-BAD probe [82]	Nicotinamide adenine dinucleotide-binding proteins	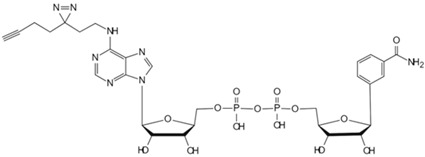
Choline	Photocrosslinkable choline probe [83]	p32	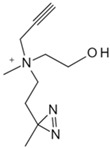
Bile acids	Clickable photoaffinity Bile acids probe [84]	EnvZ	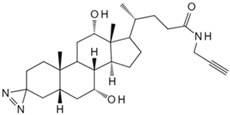
Short-chain fatty acids	Alkyne butyrate analog probe [85]	HilA	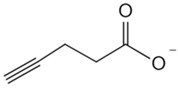
Indole-3-acetic acid	Indole-3-acetic acid analog probe [86]	G Protein-coupled receptor class C group 5 member A (GPRC5A)	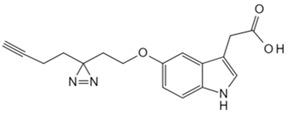
Tryptamine	Tryptamine analog probe [86]	G Protein-coupled receptor class C group 5 member A (GPRC5A)	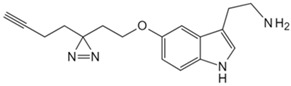

**Table 3:** Probe-free based target identification of small metabolite molecules.

Small Molecule	Method	Target
Acetyl-CoA	CoA/AcetylTraNsferase Interaction Profiling (CATNIP) [87]	Acetyl-CoA-binding proteins
Lactate	Thermal proteomic profiling (TPP) [88]	Ubiquitin conjugating enzyme E2 C (UBE2C)
Glycolysis metabolite group	Target-responsive accessibility profiling (TRAP) [61]	Glycolysis metabolite-binding protein
